# Effects of vacuum packaging in freezer on oxidative spoilage indexes of fish *Lethrinus atkinsoni*

**DOI:** 10.1002/fsn3.1704

**Published:** 2020-06-14

**Authors:** Ali Aberoumand, Farideh Baesi

**Affiliations:** ^1^ Msc in Fisherie Behbahan Khatam Alanbia University of Technology Behbahan Iran

**Keywords:** free fatty acids, Frozen *L. atkinsoni*, peroxide value, thiobarbituric acid, Vacuum

## Abstract

The improved atmosphere within the packages with low o_2_ concentration, and high concentration of co_2_ has been shown to significantly long time the shelf life of suitable fish at freezer. Vacuum packing is known to be as one of the methods of extending the shelf life of seafood. The purpose of the present study is to scrutinize the effect of vacuum packaging on oxidative spoilage indexes in fish *Lethrinus atkinsoni* fillet under freezing at −18°C in 0, 20, and 40 days. To this end, it was initially purchased 20 kg fish from a conventional market in Behbahan, Khuzestan Province, Iran. The total remaining fish after washing and waste removal was divided into packages of 150 g as the required samples and kept in a freezer. The fillets were divided into two groups: The first group was packed in polyethylene bags under vacuum, and the other group was considered as a case of treatment control, kept in freezer at −18°C. The results showed progress spoilage indexes in samples packed in vacuum during 0, 20, and 40 days found lower than control treatment with significant different (*p* < .05). The peroxide and thiobarbituric acid and free fatty acids contents decreased in frozen samples in 20 and 40 days in vacuum, but pH value increased. The results showed packed samples in freezing and in vacuum conditions, were a suitable method for low lipid oxidation in the *L. atkinsoni* fillets which led to the samples’ extended shelf life. It is concluded that packaging in vacuum as combined with freezing treatment prevails over freezing in individual by an acceptable long‐term fish shelf preservation.

## Graphical abstract

The results showed progress spoilage indexes in samples packed in vacuum on 0, 20, and 40 days with three replications were slower than in control treatments with significant effects.

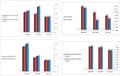

## 1 INTRODUCTION

Fish and sea food products were considered as a food source, containing high‐quality protein, vitamins, minerals, and essential and good quality fat content. The *Lethrinus atkinsoni* was found as an acceptable fish possessing high level of nutrients. This type of fish can be found in Iran (Nejad, & Kamel, 2012). It is commonly sold as whole in the Behbahan market, Kuzestan, Iran. However, because of fish spoilage, various methods have been applied to preserve fish and seafood products (Khanipur, Fathi, Fahim Dagban, & Zare, 2014). In recent years, packaging in vacuum with freezing has been found as a suitable way for the purpose of seafood preservation (Masniyom, Benjama, & Maneesri, 2013; Sahoo & Kumar, 2005). Fish as a seafood is one of the sensitive foods to spoil and the shelf life of the seafood decreases by the effects of oxygen on the growth of aerobic spoilage bacteria and enzyme activities (Ivanović et al., 2014). The improved atmosphere within the package with low o_2_ concentration and high concentration of co_2_ and/or N_2_ has been shown to provide a significant long‐time preservation for the shelf life of suitable seafood at cold temperatures (Ivanović et al., 2015; Özoğul, Taylor, Quantick, & Özoğul, 2008). Modified atmosphere packaging (MAP) and vacuum packaging (VP), along with cold temperature, have been found as enhanced preservation techniques. MAP and VP systems could provide improved seafood shelf life and suitable organoleptic quality (Koral, Sevim, & Bekir, 2010). However, packaging without oxygen may result in fat oxidative spoilage as well as improved organoleptic properties in fish (Jeveheri Baboli, Velayat Zadeh, Jagher, & Pashaei, 2016). VP is one of the various ways for a suitable preservation in order to prevent the spoilage and extend the quality of the seafood to a long time (Chaijan, 2011). VP is widely used as a supplement to freeze and decrease the supply of oxygen for the aerobic bacteria in the flesh in order to extend the shelf life of fish product (Ayala et al., 2011). The packaging process in the absence of o_2_ and in cold storage is a way to prolong the shelf life (Ježek & Buchtová, 2007). The oxygen can lead to microorganisms and photolytic activities in packed fish fillet without vacuum (Oğuzhan & Angiş, 2012; Ravi‐Sankar, Lalitha, JoseL Manju, & Gopal, 2008). Although packaging without oxygen could stop chemical changes of seafood; however, the reported data reveal that the chemical changes of the fish *Lethrinus atkinsoni* fillets, as packed under vacuum packaging, are marginal and hence may be disregarded.

The present research project is devoted to scrutinize the shelf life of the fish *Lethrinus atkinsoni* packed under vacuum packaging during frozen storage by evaluation of oxidative spoilage indexes.

## 2 MATERIALS AND METHODS

### 2.1 Sample preparation

The fish *Lethrinus atkinsoni* was purchased (20 kg) from Dilam Port, South Iran (located nearby Persian Gulf) The fish samples were placed in boxes encapsulated by ice and transferred to the Laboratory of Food for processing purpose Sari University of Technology, Iran. Fish samples were beheaded, gutted and filleted by knife, and washed with distilled cold water carefully, with the weight of each fillet being 150 ± 5 g. Shortly after, fillets were divided into two groups. Samples of the first group were marked as control treatment and packaged directly in polyethylene bags. Fillets belonging to the second group were packaged under vacuum condition in separate polyethylene bags. All packaged samples quick‐freezed to −24°C, swift enough, so as to preserve its nutritional value. After 20 hr, all fish fillets were placed in a‐18°C freezer. For all of fish fillets, analysis was carried out after the freezing process (0‐day, 20 days, and 40 days storage at −18°C). For each sample, it was considered three different repetitions and the observations were analyzed so as to provide statistical reports.

### 2.2 Method of measurement of free fatty acids

For the measurement of FFA 25 cc of neutralized ethyl alcohol, with NaOH was added to the oil sample (due to evaporation of residual solvent in the low phase of the decanter). The amount of FFA was determined in terms of the amount of normal NaOH consumed during titration in the presence of phenolphthalene and with a percentage of oleic acid (Egan, Sawyer, & Pearson's, 1997).

### 2.3 Measurement of thiobarbituric acid (TBA)

Five gram of the fish fillet with 100 ml of 10% TBA solution in a 250 ml dish was completely homogenized by electric shaker and then the homogenized solution was passed through Whitman No.42 filter paper and the filtered solution was suspended in 100 ml volume with 10% TBA solution. 3 ml of filtered solution with 3 ml of 0.02 M TBA solution was mixed in a test tube and incubated in oven at 100°C for 45 min. After cooling the samples, the optical absorption of the samples at 532 nm was measured by spectrophotometer (Ojagh, Rezaei, & Khorramgah, 2009). To prepare the control sample, 3 ml of 10% TBA solution was mixed with 3 ml of 0.02 M TBA solution and the optical absorbance of the solution was read at 532 nm. Then, the following formula was used to measure the amount of malondialdehyde per kg of the fillet (Ojagh et al., 2009).

TBA (mg malondialdehyde per kg of the fish fillet) = 50 × A_s_‐A_b_/200

A_S_ = optical absorption rate of samples

A_b_ = optical absorption rate of standard TBA solution

To prepare a standard solution of 0.02 molar TBA, 200 mg of TBA powder was dissolved in 100 ml of 5% TBA solution.

### 2.4 PH measurement method

To measure the associated pH. 5 g of the fish fillet was homogenized with 45 ml distilled water in a 250 ml dish with an electric mixer. The pH of the samples was then measured using a 713Metrohm digital pH meter (Fan, Chi, & Zhang, 2008). It should be pointed out that it was carried out three replicates for each experiment.

### 2.5 Peroxide number (PV) Method

In order to measure the peroxide value, it was extracted approximately one gram of the fish oil and further weighted in a test tube. This sample was later accompanied by the addition of one gr of potassium iodide as well as 20 ml of acetic acid and chloroform.

The test tube was placed in a boiling water beaker and boiled for about 30 seconds. Then, in Erlenmeyer, 20 cm^3^ of 5% potassium iodide solution and a few drops of 1‐5% starch were added. Afterward, the test tube was washed twice with 25 cc of water and added to Erlenmeyer with sodium hyposulfite solution 1.5 N. for titration. The peroxide content is calculated in milli eq of oxygen per kg of the sample as follows: (AOAC., 2005).

PV value = Normality × Consumption volume of sodium hyposulfite/Sample weight of oil × 1,000.

### 2.6 Statistical analysis

Data were analyzed using analysis of variance (ANOVA). The least significant difference procedure was applied to test for differences between means. Statistical analysis was carried out with SPSS 16.0.

## 3 RESULTS AND DISCUSSION

**FIGURE 1:**
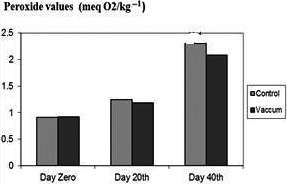
Comparison of peroxide values of control and treatments

The peroxide value shows that the initial level of fat oxidation products was significantly lower (*p* < .05) for packaging in vacuum at the beginning of the research than that of for simple packaging (2.12 ± 0.13 and 2.35 ± 0.03 meq O_2_/kg‐1, respectively). Differences (*p* < .05) between the two types of packaging were also found at the days of 20 and 40. But the PV content for packaging in vacuum increased during the research from 1.16 ± 0.07 meq O_2_/kg‐1 to 2.12 ± 0.13 (*p* < .05); this is while the PV for simple packaging increased significantly from 1.29 ± 0.08 to 2.35 ± 0.03 (*p* < .05). The PV values at the end of the period were very similar (see Figure 1).

**FIGURE 2:**
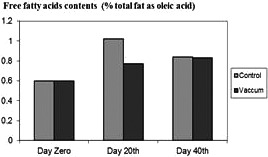
Comparison of free fatty acids contents of control and treatments

**FIGURE 3:**
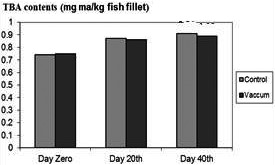
Comparison of TBA contents of control and treatments

However, the FFA contents (% total fat as oleic acid) for packaging in vacuum in the fish muscle increased significantly from 0.62 ± 0.01 to 0.81 ± 0.05 between days 0 and 40. It is to some extent mentioning that in simple packaging, the difference was not significant (referred to Figure 2). TBA values in the samples with different conditions are shown in Figure 3.

An increase in TBA content was found in all samples as the storage time was extended (*p* < .05), indicating, in particular, that lipid oxidation occurred during storage (Figure 3).

**FIGURE 4:**
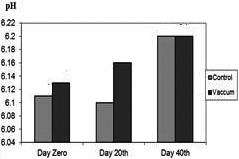
Comparison of pH values of control and treatments

The pH values decreased in the *Lethrinus atkinsoni* fillet of packaged in vacuum were different from samples in simple packaging. The initial pH values (day 0) in packaged in vacuum were significantly (*p* < .05) higher (6.15 ± 0.02) than those in simple packaging (6.13 ± 0.05). However, the pH values associated with packaged in vacuum increased until day 40 (6.21 ± 0.02), pH values in simple packaging decreased until day 20 (6.12 ± 0.02). At the end of the period (day 40), pH values in both types of packaging were found identical (see Figure 4).

The differences found between the packaging in vacuum and simple packaging were not significant in view of statistical analysis (*p* < .05). The fats in fillets contain high percentage of unsaturated fatty acids and are therefore sensitive to the reaction of oxidative spoilage. (Ježek & Buchtová, 2014). However, packaged sample in vacuum showed the higher TBA content, compared with samples stored in a casual manner. This showed that packaging in vacuum could prevent oxidative spoilage and improve organoleptic quality of fish. The result was found in agreement with Gimenez, Roncales, and Beltrán (2002).

The increase of FFA in *Lethrinus atkinsoni* fillets could be linked to the lipase and phospholipase activity in *Lethrinus atkinsoni* fillets during frozen storage. Analogous results have been reported by other researchers (Rostamzad, Sha'banpour, Shabani, & Kashani Nejad, 2009). PV and TBA contents are related to fat degradation.

The peroxide content, whenever it increases, serves as an indication of the expiration of the shelf life of sample. The main application of a PV is pertinent to the quality of oil samples (Shamloofar, Hosseini, Kamali, & Matlebi, 2013). The presence of TBA in a sample of fillet shows that lipid peroxidation has occurred. The level of TBA shows the amount of peroxidation, already occurred (Etemadian, Shabanpour, Sadeghi Mahounk, Shabani, & Dordai, 2012). Rostamzad et al. (2009) and Anelich, Hoffman, and Swanpoel (2001) reported lower TBA values in samples which were treated by vacuum packaging as compared with control samples. The increase in TBA showed formation of secondary lipid oxidation products such as aldehydes and other volatiles compounds.

Comparison of PV, TBA, and FFA values in control and storage samples in vacuum at the day 40th showed significant difference (*p* < .05), while there was no significant difference at the day 20th as well as at the day 0 (*p* < .05). There was no significant difference between all items in control and storage samples in vacuum for pH (*p* < .05) (see Table 1). Packaging in vacuum has been found to substantially reduce oxidative deterioration in frozen fish (Masniyom et al., 2013), as agreed with the present reports.

**TABLE 1:** Indexes of spoilage of fish *Lethrinus atkinsoni* fillet packed in two methods of vacuum and typical in freezing periods

Indicators	Day zero		Day 20th		Day 40th	
Freezing period	Control	Vacuum	Control	Vacuum	Control	Vacuum
PV	0.92 ± 0.01^a^	0.93 ± 0.01^a^	1.29 ± 0.08^a^	1.16 ± 0.07^a^	2.35 ± 0.03^a^	2.12 ± 0.13^b^
FFA	0.62 ± 0.02^a^	0.62 ± 0.01^a^	1.04 ± 0.44^a^	0.74 ± 0.05^a^	0.86 ± 0.01^a^	0.81 ± 0.05^b^
TBA	0.75 ± 0.01^a^	0.76 ± 0.01^a^	0.88 ± 0.01^a^	0.87 ± 0.01^a^	0.92 ± 0.05^a^	0.90 ± 0.05^b^
pH	6.13 ± 0.05^a^	6.15 ± 0.02^a^	6.12 ± 0.02^a^	6.17 ± 0.03^a^	6.20 ± 0.01^a^	6.21 ± 0.02^a^

Note

The same letters in each raw indicate a nonsignificant difference (*p* < .05).

Abbreviations: FFA, free fatty acids; PV, peroxide value; TBA, thiobarbitoric acids.

Balev, Staykov, Ivanov, Dragoev, and Filizov (2011) reported that at the end of storage, the total FFA concentration of air packaged, and vacuum packaged samples were found as 1.17 and 0.85 g/kg fresh fish weight, respectively, in Russian Sturgeon during frozen storage. Their results showed that vacuum packaging significantly (*p* < .05) delayed lipolysis of lipid. This result was in agreement with the present observations. It was concluded that vacuum packaging treatment has significant effects on delaying lipid oxidation. In vacuum packaging at the 40th‐day treatment (see Figure 4), constant level of pH might be attributed to the increasing solubility of CO_2_ at storage time, affecting on growth of aerobic microflora, (Taheri, & Motallaabi, 2012). Variations in pH values in the fish fillet during frozen storage are duo to the destroying changes of compounds, containing high energy and protein at the end of the period; this is as well as conversion of CO_2_ to carbonic acid leading to a decrease in fillet pH values. The pH values of the studied fish fillet were compared with the upshots by other researchers (Arashisar, Hisar, Kaya, & Yanik, 2004; Chytiri, Chouliara, Savvaidis, & Kontominas, 2004; Gimenez et al., 2002; Jasour, Rahimabadi, Ehsani, Rahnama, & Arshadi, 2011; Masniyom, 2011). The fat hydrolysis resulted into increase of FFA. The FFA contents were not significantly affected by packaging in vacuum except for 20‐day treatment (see Figure 2). The procured values did not agree with those obtained by other researchers. An increasing amount of FFA is often related to a loss of freshness in fish fillet. The release of FFA is perhaps in connection with the oxidation of lipids, causing off‐flavor and off‐odor (Rezaei, Hosseini, Langrudi, Safari, & Hosseini, 2008). Some authors (Bahmani,et al., 2011; Özyurt, Kuley, Özkütük, & Özogul, 2009) believe FFA formation is related to the loss of freshness in fish and, consequently, significant effects on the sensory quality of fish. Fagan, Gormley, and Uí Mhuircheartaigh (2004) showed that FFA has no impacts on organoleptic properties. Lipid oxidation leads to a shorter shelf life in fish. The peroxide value (PV) was used to determine hydroperoxides. Significant fluctuations in PV were recorded in this study, particularly in simple packaging. Similar fluctuations have also been found by other authors (Rezaei et al., 2008). Initial PV is an index for the occurrence of oxidation during handling and processing, and a subsequent decline in PV is then caused by reactions that lead to increase in TBA contents (Chaijan, 2011). The TBA value may not show the main reason of oxidation, followed by the fact that malondialdehyde can interact with other components, being the ending products of lipid oxidation. These interactions vary with respect to different fish species (Chytiri et al., 2004). The TBA value can be recommended as a suitable index for oxidative changes. According to the present findings, vacuum packaging can be recommended as an appropriate protection against fish fillet oxidation.

Aberoumand, Ziaei Nejad, Baesi, and Kolyaee (2018) studied the effects of freezing on the physical and chemical properties of the fillet of some fish species. The obtained results showed that the TBA content in fish fillet for *P. argenteus* and *S. hasta* was 0.65 and 0.53, respectively. The highest percentage of fat was found for *P. argenteus* after 95 days (24.22%), and for *S. hasta* after 35 days (25.19%). The highest free fatty acid contents (0.9% and 0.97%) were found for *S. hasta* and *P. argenteus* after 95 days.

Freezing and frozen storage affected the FFA, TBA, and pH. An increase in the contents of FFA, TBA, and pH in the fish fillet was observed as compared to the control. A statistical analysis revealed that there was no significant difference between TBA acid changes in the fish *S. hasta* in freezing periods; however, a significant difference was found in the TBA content in the fish *P. argenteus*. The PV in *P. argenteus* increased between free fatty acids and pH contents in the fillets of *P. argenteus* and *S. hasta*, and significant differences were observed. This study has shown low‐fat oxidation and rancidity in the fillet of the fish *P. argenteus* in a freezing period as compared to the control. It can be concluded that the TBA and FFA contents and pH level of two fish species are high during frozen storage. The PV in *P. argenteus* increased, but in *S. hast*a showed no significant difference. The best time of frozen storage of *P. argenteus* and *S. hasta* was at 35 days, but the nutritional value of the fillet and the FA increased greatly.

## 4 CONCLUSIONS

The effects of vacuum packaging were to delay lipid oxidation which was studied on the *Lethrinus atkinsoni* fillets. Results showed that frozen storage period up to 40 days decreased FFA, PV, and TBA contents. According to the present results, packaging samples under freezing and vacuum conditions are a suitable route for reducing lipid oxidation of the *Lethrinus atkinsoni* fillets and, consequently, extending the shelf life.

## CONFLICTS OF INTEREST

The authors do not have any conflict of interest.

## ETHICAL REVIEW

This study did not involve human or animal testing.

## INFORMED CONSENT

Written informed consent was obtained from all study participants.
